# Analysis of potential genetic biomarkers using machine learning methods and immune infiltration regulatory mechanisms underlying atrial fibrillation

**DOI:** 10.1186/s12920-022-01212-0

**Published:** 2022-03-19

**Authors:** Li-Da Wu, Feng Li, Jia-Yi Chen, Jie Zhang, Ling-Ling Qian, Ru-Xing Wang

**Affiliations:** Department of Cardiology, Wuxi People’s Hospital Affiliated to Nanjing Medical University, No. 299, Qingyang Road, Wuxi, 214023 China

**Keywords:** Atrial fibrillation, Immune infiltration, Biomarker, CIBERSORT, Diagnostic biomarker

## Abstract

**Objective:**

We aimed to screen out biomarkers for atrial fibrillation (AF) based on machine learning methods and evaluate the degree of immune infiltration in AF patients in detail.

**Methods:**

Two datasets (GSE41177 and GSE79768) related to AF were downloaded from Gene expression omnibus (GEO) database and merged for further analysis. Differentially expressed genes (DEGs) were screened out using “*limma*” package in R software. Candidate biomarkers for AF were identified using machine learning methods of the LASSO regression algorithm and SVM-RFE algorithm. Receiver operating characteristic (ROC) curve was employed to assess the diagnostic effectiveness of biomarkers, which was further validated in another independent validation dataset of GSE14975. Moreover, we used CIBERSORT to study the proportion of infiltrating immune cells in each sample, and the Spearman method was used to explore the correlation between biomarkers and immune cells.

**Results:**

129 DEGs were identified, and *CYBB*, *CXCR2*, and *S100A4* were identified as key biomarkers of AF using LASSO regression and SVM-RFE algorithm. Both in the training dataset and the validation dataset, *CYBB*, *CXCR2*, and *S100A4* showed favorable diagnostic effectiveness. Immune infiltration analysis indicated that, compared with sinus rhythm (SR), the atrial samples of patients with AF contained a higher T cells gamma delta, neutrophils and mast cells resting, whereas T cells follicular helper were relatively lower. Correlation analysis demonstrated that *CYBB*, *CXCR2*, and *S100A4* were significantly correlated with the infiltrating immune cells.

**Conclusions:**

In conclusion, this study suggested that *CYBB*, *CXCR2*, and *S100A4* are key biomarkers of AF correlated with infiltrating immune cells, and infiltrating immune cells play pivotal roles in AF.

**Supplementary Information:**

The online version contains supplementary material available at 10.1186/s12920-022-01212-0.

## Introduction

As the most common arrhythmia in clinic, patients with atrial fibrillation (AF) have high mortality and morbidity. It is reported that about 1–2% of the population are troubled by AF, which contributes to heart failure and cardiogenic embolism [1]. AF could be divided into permanent AF, persistent AF (pAF), long standing pAF and paroxysmal AF based on its duration. Patients with hypertension, obesity, and diabetes etc. frequently develop AF, however, the molecular mechanisms underlying the development of AF remain unclear yet [2]. Inflammatory response plays an important role in the occurrence and development of AF. Studies have shown that TNF-α, CRP and IL -6 are significantly increased in atrial tissues of AF patients and related to outcomes of AF patients [3–5]. Moreover, studies demonstrated the anti-inflammatory therapies can significantly reduce AF episodes [6, 7]. In recent years, the role of immune cells infiltration in the inflammatory response of patients with AF has been widely concerned. Yamashita et al*.* confirmed that, in human AF, adhesion and recruitment of macrophages in heart endocardium promoted inflammatory responses [8]. Similarly, Hohmann et al*.* demonstrated that the number of CD3-positive T cells in left atrial appendageal are significantly increased in patients with AF [9]. However, the more accurate association between infiltrating immune cells and AF still needs to further study.

Medicine is one of the early applications of artificial intelligence (AI), which is gradually changing the way many diseases are diagnosed and treated [10]. Machine learning is an important part of artificial intelligence that using algorithms to identify expression patterns of datasets. Machine learning has already been employed in prediction of myocardial infarction, pathological identification and surgical improvement [11]. Moreover, machine learning is also a research hotspot and cutting-edge technology in the field of arrhythmia diagnosis and treatment. Han et al*.* used machine learning algorithms to incorporate clinical signatures of AF patients, and their work provides prognostic value for risk stratification in stroke beyond CHA2DS2-VASc [12]. The diagnosis of atrial fibrillation requires electrocardiogram (ECG) test, but some asymptomatic patients are often difficult to find. Raghunath et al*.* collected 12 lead ECGs of 430,000 patients and predicted new onset atrial fibrillation within 1 year based on deep neural network. It was found that the accuracy of the algorithm reached 0.85 [13]. To the best of our knowledge, we firstly conducted a bioinformatics analysis to screen out key differentially expressed genes (DEGs) in AF as biomarkers based on machine learning algorithms. The CIBERSORT algorithm has been adopted to evaluate infiltrating immune cells based on gene expression profiles in various diseases [14–17]. We also performed a detailed analysis of immune cells infiltration in patients with AF using CIBERSORT algorithm.

## Materials and methods

### Microarray data

**Fig. 1:**
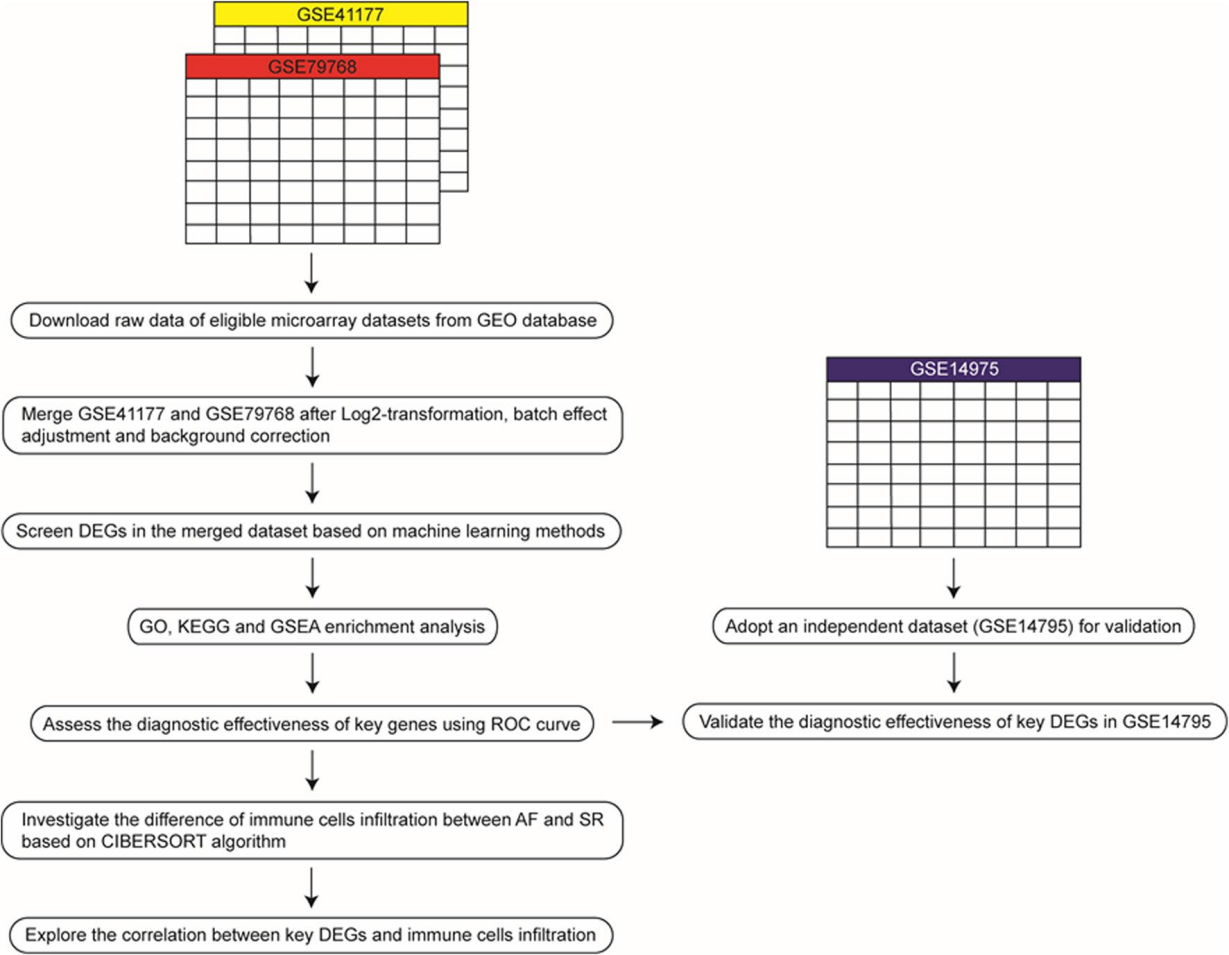
Workflow of data preparation, processing and analysis. GEO, Gene Expression Omnibus; DEGs, differentially expressed genes; AF, atrial fibrillation; SR, sinus rhythm; GSEA, gene set enrichment analysis

The workflow of this analysis is shown in Fig. 1. Three datasets related to AF (GSE41177, GSE79768 and GSE14975) were downloaded from Gene Expression Omnibus (GEO) database [18] via “*GEO query*” package [19]. The above three datasets were all based on GPL570 platform. GSE41177 contained 19 left atrial tissue samples from 3 SR individuals and 16 AF patients [20]; GSE79768 consisted of 13 left atrial tissue samples from 7 AF patients and 6 SR individuals [21]; GSE14975 contained 10 left atrial tissue samples from 5 SR individuals and 5 AF patients [22]. The detailed characteristics of GSE41177, GSE79768 and GSE14975 is provided in Additional file 2: Table S1.

### Data processing and DEGs screening

R software was employed to create gene expression matrices of GSE41177, GSE79768 and GSE14975. Log2-transformation and background correction were performed on the expression profiles by the “*limma*” package [23]. Furthermore, “*SVA*” package was used for batch effects adjustment between the GSE41177 and GSE79768 [24]. GSE41177 and GSE79768 were merged for further analysis, and the GSE14795 was used as the validation cohort. “*pheatmap*” package and “*ggplot2*” package was adopted to create to “heatmap” and “volcano plot” of DEGs.

### Enrichment analysis

To understand the function of DEGs in AF patients, the “*clusterProfler*” package was used to perform GO and KEGG pathway analysis [25]. Gene set enrichment analysis (GSEA) was also employed to identify pathways enriched in AF patients and SR individuals, respectively. “c2.cp.kegg.v7.0.symbols.gmt” from MSigDB database was adopted as the reference dataset [26].

### Identification of key DEGs as biomarkers in AF using machine learning methods

Machine learning methods were adopted to screen out key DEGs as biomarkers in AF. LASSO algorithm, a regression analysis, often utilized to improve prediction accuracy. It belongs to linear regression model family and uses the default ten-fold cross validation. In recent years, LASSO regression analysis has been widely used in researches to screen out diagnostic or prognostic factors [27]. Jubair et al*.* found a meaningful way to identify subtype‑specific biomarkers for the breast cancer survivability using LASSO regression analysis [28]. Ma et al*.* also identified key genes in blood of patients with intervertebral disc degeneration (IDD) as important biomarkers based on LASSO regression analysis [29]. To screen out key genes correlated with AF, *“glmnet”* package was used to perform LASSO regression algorithm. SVM-RFE is another machine learning algorithm, which has been widely used for classification and regression analysis. SVM-RFE model has nonlinear discrimination characteristics, which allows the results to be compared after modeling different numbers of variables, so as to screen the best combination of variables. Based on SVM-RFE algorithm, Zhang et al*.* screened ten discriminant features, which provided a fast and effective diagnostic standard for Kashin–Beck disease [30]. We also used “*e107*” package to carry out SVM-RFE algorithm and identify key genes in occurrence and development of AF with discriminative power [31].

### Diagnostic value of key DEGs as biomarkers in AF

Receiver operating characteristic (ROC) curve was established based on the meta-data cohort merged by GSE41177 and GSE79768 to evaluate the predictive value of biomarkers. We used the area under curve (AUC) value to determine the diagnostic effectiveness in discriminating AF from SR patients. Then, an independent dataset (GSE14975) was adopted to further validate the diagnostic effectiveness of biomarkers.

### Evaluation of infiltrating immune cells

CIBERSORT algorithm was employed to evaluate infiltrating immune cells in patients with AF [17]. A large number of studies have used CIBERSORT to explore the function of immune cells in various diseases, including osteoarthritis [14], high-grade serous ovarian cancer [15] and breast ductal and lobular carcinoma [16]. Proportions of infiltrating immune cells were visualized in R software using “*ggplot2*” package and “*pheatmap*” package. Correlation heatmap was created by “*corrplot*” package to visualize the correlation of infiltrating immune cells. The difference of immune cells infiltration between atrial tissue samples from AF patients and SR individuals were showed in the violin plot using the “*vioplot*” package. Then, “*ggplot2*” package was also adopted to perform principal components analysis (PCA) based on immune cells infiltration and draw a two dimensional PCA plot.

### Correlation analysis of biomarkers and infiltrating immune cells

We used “Spearman” method to explore the correlation between biomarkers and immune infiltration, and then we used the “*ggplot2*” package to visualize results.

## Results

### Identification of DEGs in AF

**Fig. 2:**
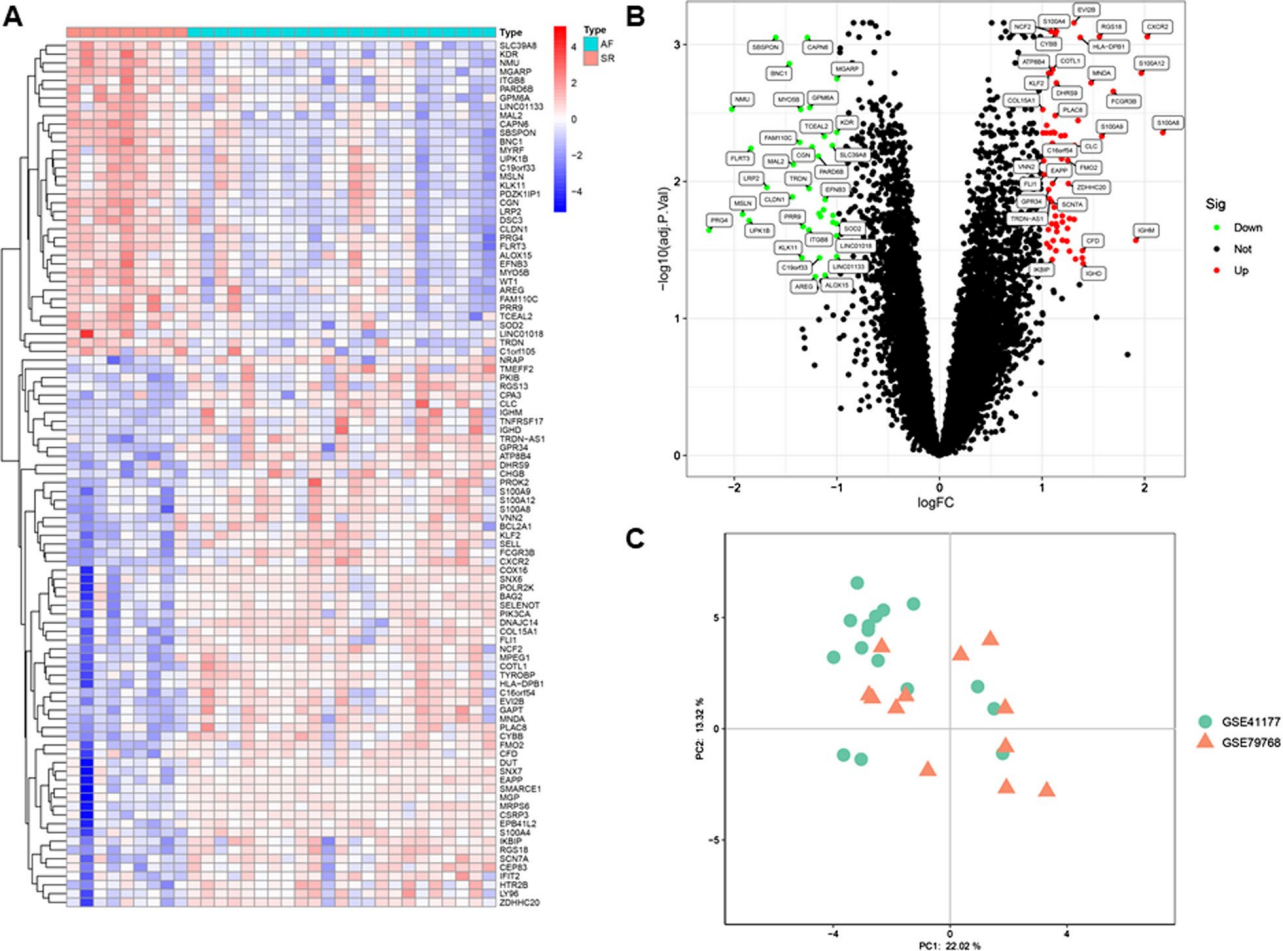
Identification of DEGs between AF and SR atrial tissue samples. **a** Heatmap visualization of the top 100 DEGs between AF and SR atrial tissue samples. **b** Volcano plot visualization of DEGs between AF and SR atrial tissue samples. **c** PCA plot of AF and SR atrial tissue samples after removing batch effect between GSE41177 and GSE79768. AF, atrial fibrillation; SR, sinus rhythm; DEGs, differentially expressed genes

Left atrial tissues from 23 AF patients and 9 normal individuals of GSE41177 and GSE79768 were analyzed. PCA plot clearly indicated that the batch effect between GSE41177 and GSE79768 was successfully removed (Fig. 2c). In the meta-data cohort merged by GSE41177 and GSE79768, 129 DEGs were identified using the *“limma”* package, including 71 upregulated genes and 58 downregulated genes (Fig. 2a, b).

### Functional correlation analysis

**Fig. 3:**
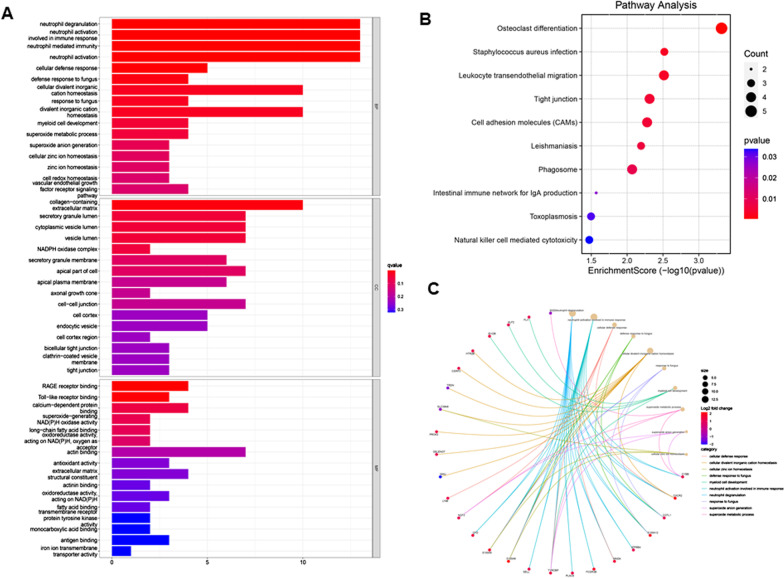
Enrichment analysis of DEGs between AF and SR atrial tissue samples via GO and KEGG database. **a** Gene ontology enrichment analysis of DEGs. **b** KEGG enrichment analysis of DEGs. **c** Cord diagram shows the relationship between key DEGs and most enriched biological processes. AF, atrial fibrillation; SR, sinus rhythm; DEGs, differentially expressed genes

**Fig. 4:**
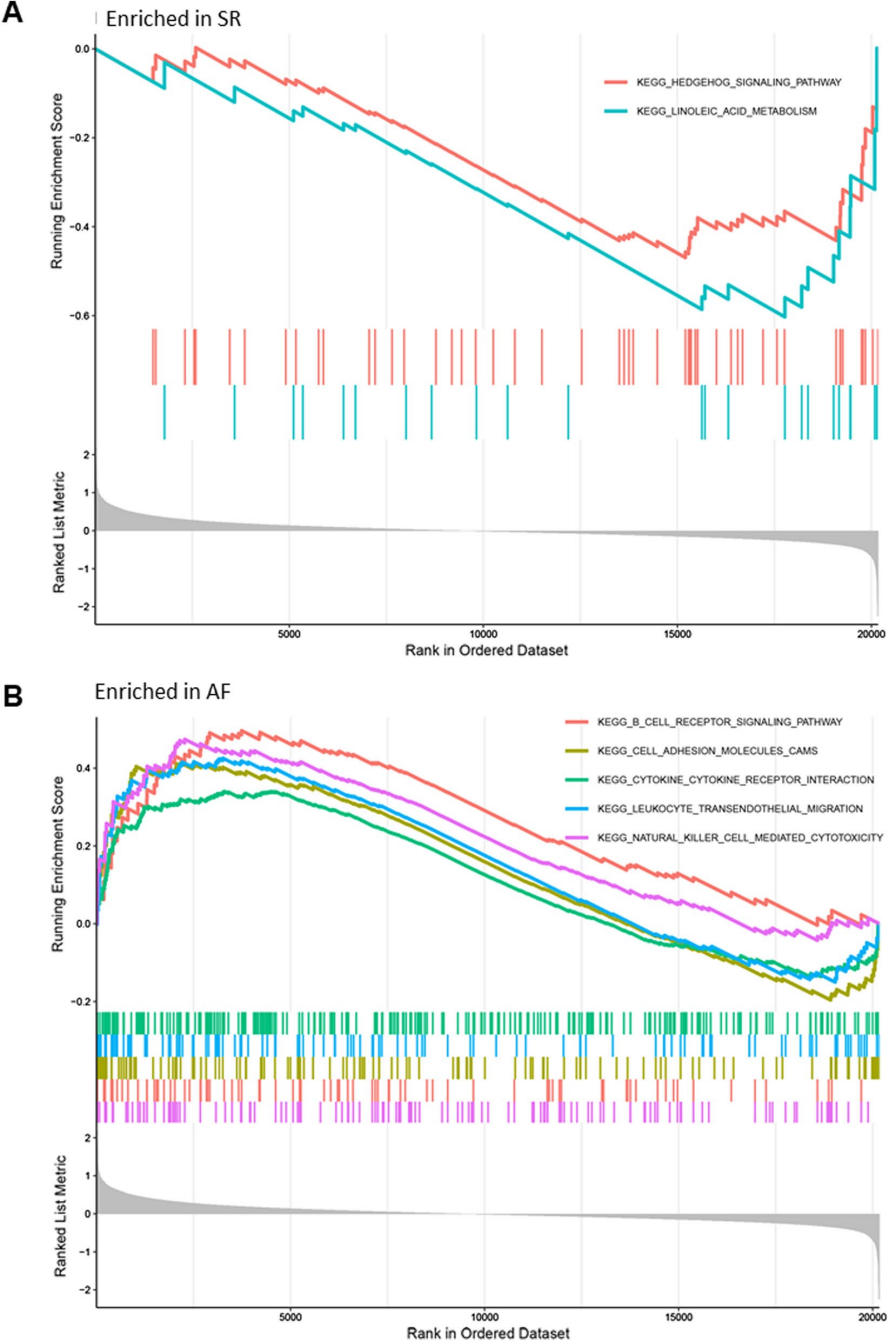
GSEA enrichment analysis of DEGs between AF and SR atrial tissue samples. **a** GSEA enrichment analysis results in SR patients. **b** GSEA enrichment analysis results in AF patients. AF, atrial fibrillation; SR, sinus rhythm; GSEA, gene set enrichment analysis; DEGs, differentially expressed genes

We performed functional enrichment analysis of DEGs between AF and SR patients based on GO and KEGG databases (Fig. 3a). The biological processes were enriched in neutrophil activation involved in immune response, neutrophil mediated immunity, neutrophil degranulation, neutrophil activation and cell cellular defense response. The relationship between biological processes terms and each DEG was shown in Fig. 3c. The enriched cellular components were mainly involved in collagen-containing extracellular matrix, secretory granule lumen, cytoplasmic vesicle lumen, vesicle lumen and NADPH oxidase complex. The molecular functions were mainly enriched in RAGE receptor binding, Toll-like receptor binding, calcium-dependent protein binding, superoxide-generating NADPH oxidase activity and long-chain fatty and binding oxidoreductase activity. KEGG pathway analysis shows that the osteoclast differentiation, staphylococcus aureus infection, leukocyte trans-endothelial migration, tight junction and cell adhesion molecules were mostly enriched (Fig. 3b). Moreover, GSEA results showed that Hedgehog singling pathway and linoleic acid metabolism were mainly enriched in SR (Fig. 4a). The receptor signaling pathway, cell adhesion molecules cams, cytokine-cytokine receptor interaction, leukocyte trans-endothelial migration and natural killer cell mediated cytotoxicity were mainly enriched in AF (Fig. 4b).

### Identification key DEGs as biomarkers of AF based on machine learning algorithms

**Fig. 5:**
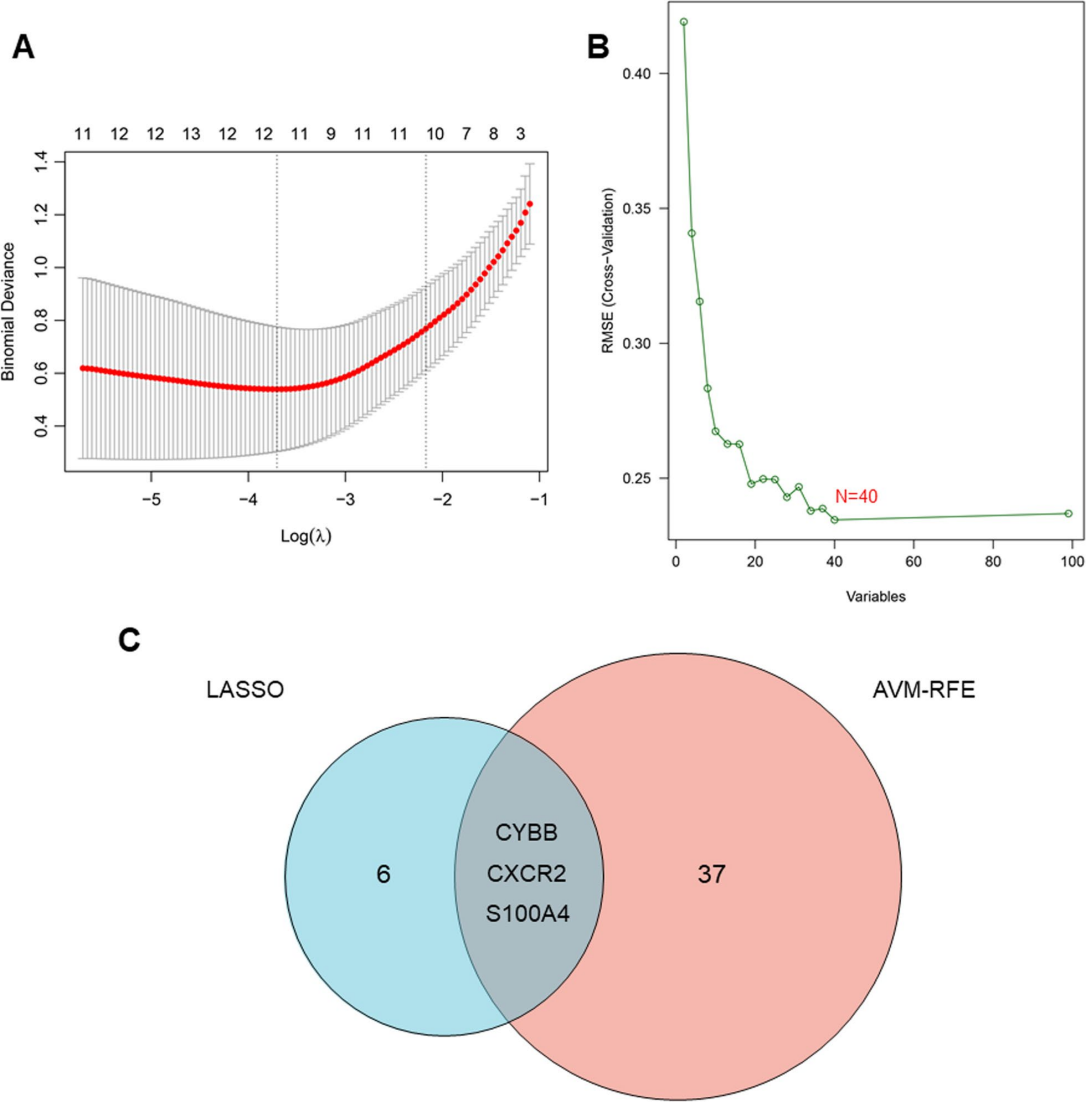
Identification of biomarker candidates for AF based on machine learning algorithms. **a** Biomarkers selection via LASSO algorithm. **b** Biomarkers selection via SVM-RFE algorithm. **c** Venn plot of the overlapping genes identified by the LASSO algorithm and SVM-RFE algorithm. AF, atrial fibrillation; LASSO, least absolute shrinkage and selection operator model; SVM-RFE, support vector machine-recursive feature elimination model

We used two different machine learning algorithms to screen key DEGs as biomarkers of AF. 9 key DEGs were identified using LASSO algorithm in the present study (Fig. 5a). Moreover, 40 DEGs was identified as biomarkers based on SVM-RFE algorithm (Fig. 5b). The three overlapping DEGs (*CXCR2*, *CYBB* and *S100A4*) were ultimately selected (Fig. 5c).

### Diagnostic effectiveness of biomarkers in AF

**Fig. 6:**
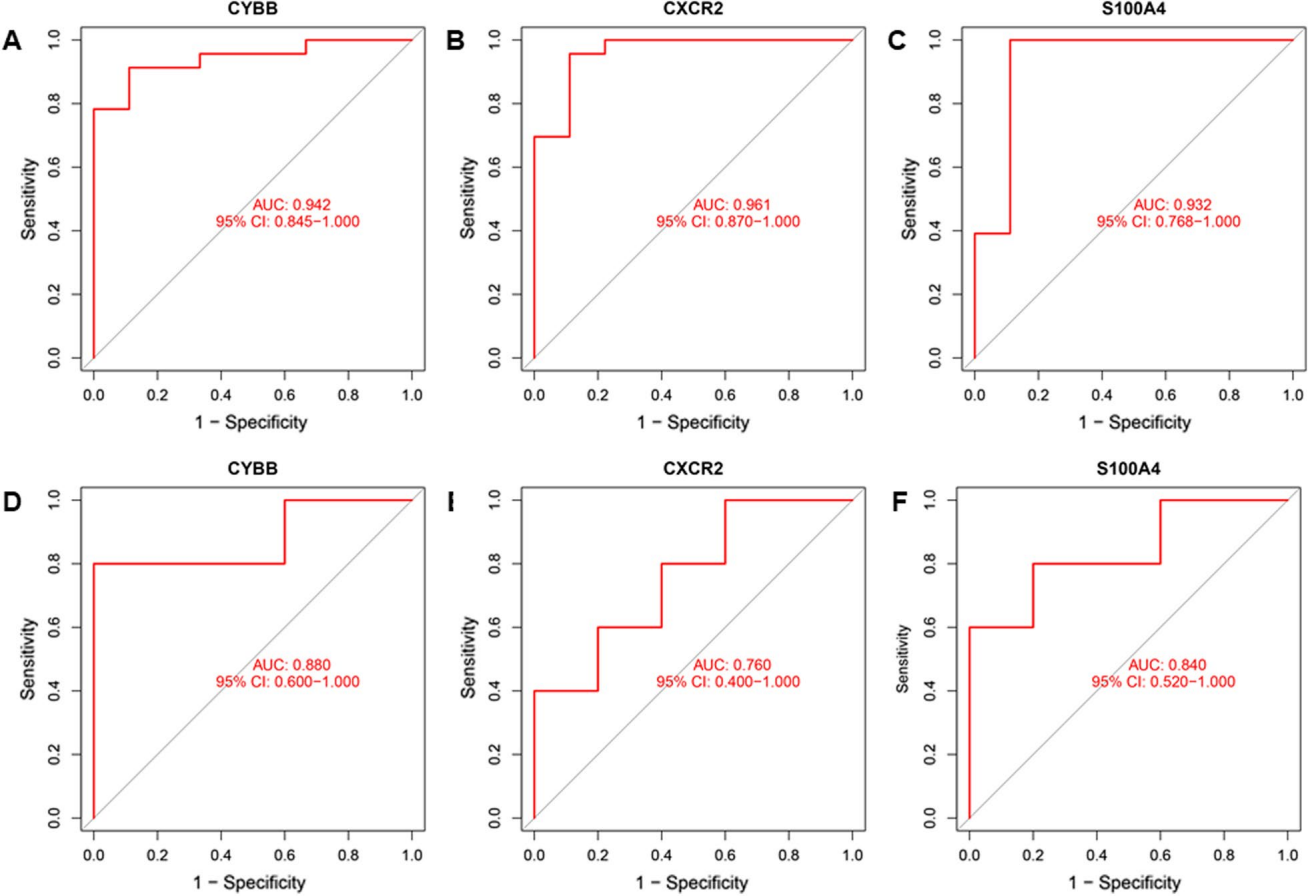
Evaluation of the diagnostic effectiveness of the three biomarkers. **a**–**c** ROC curve of *CYBB*, *CXCR2* and *S100A4* in the metadata cohort merged by GSE41177 and GSE79768; **d–f** ROC curve of *CYBB*, *CXCR2* and *S100A4* in another independent validation dataset of GSE14795. ROC, receiver operating characteristic

Our results of ROC curves indicated that these three biomarkers screened out by machine learning algorithms also have a favorable diagnostic value in the meta-data cohort merged by GSE41177 and GSE79768, with an AUC of 0.942 (95% CI 0.845–1.000) in *CYBB*, AUC of 0.961 (95% CI 0.870–1.000) in *CXCR2*, and AUC of 0.932 (95% CI 0.768–1.000) in *S100A4* (Fig. 6a–c).Moreover, the diagnostic effectiveness of key DEGs was further validated in another independent dataset (GSE14795) with an AUC of 0.880 (95% CI 0.600–1.000) in *CYBB*, AUC of 0.760 (95% CI 0.400–1.000) in *CXCR2*, and AUC of 0.840 (95% CI 0.520–0.912) in *S100A4* (Fig. 6d–f).

### Immune infiltration analysis

**Fig. 7:**
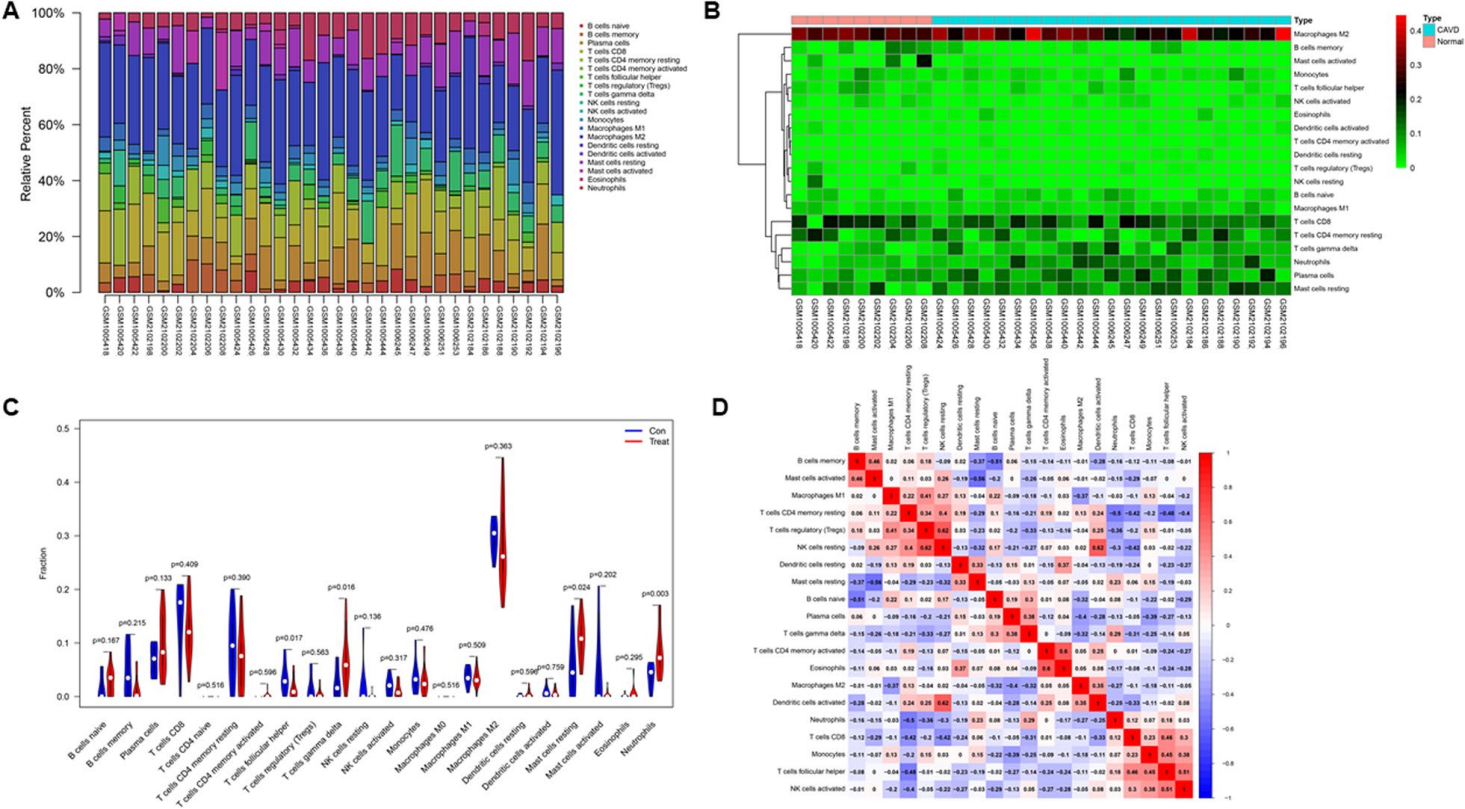
Evaluation and visualization of immune cells infiltration in AF and SR atrial tissue samples. **a** The proportion of infiltrating immune cells in AF and SR atrial tissue samples. **b** Heatmap of infiltrating immune cells in AF and SR atrial tissue samples. **c** The difference of 22 subpopulations of immune cells between AF and SR atrial tissue samples. **d** Correlation heatmap shows the correlation between 22 immune cell subpopulations. AF, atrial fibrillation; SR, sinus rhythm

Based on CIBERSORT, we evaluated immune cells infiltration in patients with AF and normal individuals. Figure 7a, b illustrate the proportion of immune cells from 9 SR left atrial tissue samples and 23 AF left atrial tissue samples. As shown in Fig. 7c, compared with SR, left atrial tissue samples from AF patients contained higher neutrophils, mast cells resting and T cells gamma delta, whereas lower T cells follicular helper. Correlation analysis showed that dendritic cells activated and NK cells resting had the most intense positive relationship with r = 0.62, B cells naïve and B cells memory had the most obvious negative correlation with r = − 0.51 (Fig. 7d). PCA diagram revealed a distinct group bias and proved that the degree of immune cells infiltration is different between AF patients and SR individuals (Additional file 1: Fig. S1).

### Correlation analysis between CXCR2, CYBB and S100A4 and infiltrating immune cells

**Fig. 8:**
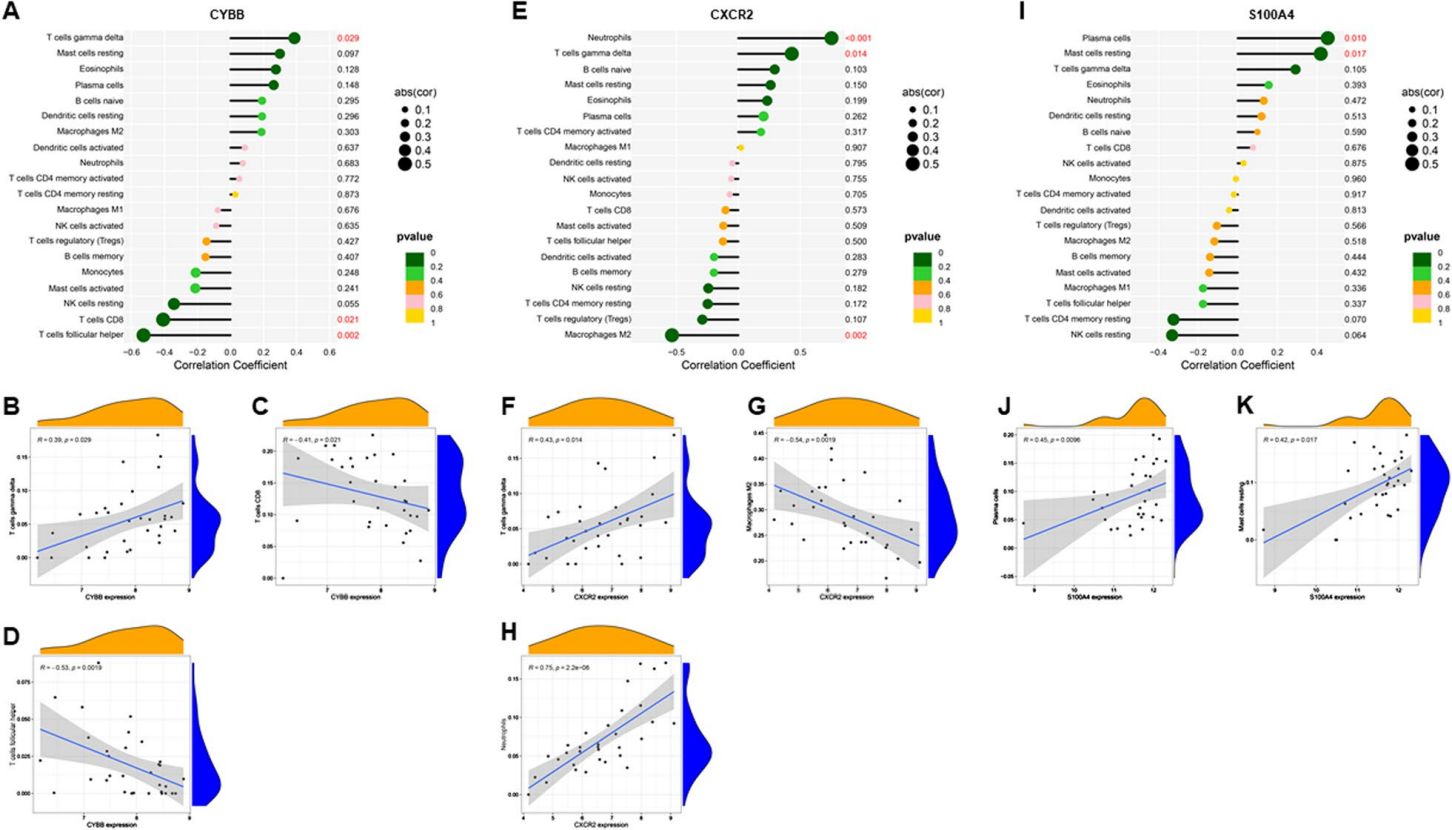
Correlations between *CYBB, CXCR2, S100A4* and infiltrating immune cells in AF. **a**–**d** Correlation between *CYBB* and infiltrating immune cells in AF. **e**–**h** Correlation between *CXCR2* and infiltrating immune cells in AF. **i**–**k** Correlation between *S100A4* and infiltrating immune cells in AF. AF, atrial fibrillation

In correlation analysis, we demonstrated that *CYBB* was positively correlated with T cells gamma delta (r = 0.28, P = 0.029) and negatively correlated with T cells CD8 (r =  − 0.41, P = 0.021), T cells follicular helper (r =  − 0.52, P = 0.002) (Fig. 8a–d). *CXCR2* was positively correlated with T cells gamma delta (r = 0.43, P = 0.014), neutrophils (r = 0.75, P < 0.001) and negatively correlated with macrophages M2 (r =  − 0.53, P = 0.002) (Fig. 8e–h). *S100A4* was positively correlated with plasma cells (r = 0.45, P = 0.01) and mast cells resting (r = 0.42, P = 0.017) (Fig. 8i–k).

## Discussion

AF is one of the most prevalent arrhythmias, however, the specific molecular mechanisms of AF still remain unclear. Despite the great improvement has been gained in the field of diagnosis and treatment, AF remains a leading cause of mortality and disability [32]. Drugs for rate control, oral anticoagulants for stroke prevention, antiarrhythmic drug and catheter ablation for conversion are main treatments for AF patients [33]. But the efficacy and safety of these treatment measures are still not well understood. Meanwhile, many asymptomatic patients, especially patients with paroxysmal AF, are difficult to find. This is the first study to identify biomarkers of AF associated with immune cells infiltration. Two gene expression datasets from GEO database were merged and conducted an integrated analysis. 129 DEGs were detected using *“limma”* package. Enrichment analysis showed that these 129 DEGs were significantly correlated with immune and inflammatory responses. The relationship between inflammatory response and AF has been widely studied in the past decades and various regular anti-inflammatory biomarkers were found be related to AF. It has been reported that patients with AF had increased IL-6, IL-8 and TNF-α, meanwhile, these inflammatory markers can also predict the outcome of AF ablation [34–38]. High-sensitive C-reactive protein (hsCRP) is also associated with increased risk of AF recurrence following successful electrical cardioversion and catheter ablation [39]. In addition, accumulating studies have demonstrated that the increase of TGFβ1 in AF patients promote atrial fibrosis, which plays a pivotal role in atrial structural remodeling in AF [40, 41]. Additionally, the critical role of various immune cells in the pathogenesis of AF has attracted more and more attention, including infiltrating in the atrium and secreting several chemokines and cytokines to regulate the microenvironment of the heart [42]. Our GSEA results are also in general agreement with the previous findings that immune cells infiltration participates in the pathogenesis of AF [8, 9]. Medicine is the earliest application field of AI. In the past few decades, AI technology, especially machine learning, has made great progress in the diagnosis and treatment of a variety of diseases, including cardiovascular diseases, nervous system diseases and genetic diseases [43]. Therefore, for the first time, we sought to screen out key DEGs between AF and SR patients as biomarkers based on machine learning methods and explore its relationship with immune cells infiltration in AF. Overlapping the results from two machine learning algorithms, *CYBB*, *CXCR2* and *S100A4* were identified as key DEGs and biomarkers of AF.

*CYBB*, also known as NOX2, has been implicated in oxidative stress in various cardiovascular diseases [44]. Pignatelli et al*.* reported that serum NOX2 can be used as one of the important indicators to predict vascular embolism events in [45]. In animal model, numerous studies have demonstrated that inhibition of NOX2-mediated production of reactive oxygen species (ROS) prevents atrial remodeling [46, 47]. In addition, atrial electrical remodeling can also be alleviated by inhibiting NOX2 and oxidative stress [47, 48]. In human AF, NOX2 has also been demonstrated to participate in the atrial structural remodeling and electrophysiological remodeling, and up-regulation of NOX2 is associated with an enhanced risk of AF [49, 50].

The chemokine receptor CXCR2, encoding by *CXCR2*, belongs to chemokine receptors family, mediates cellular migration of immune cells [51]. The expression level of *CXCR2* is tightly regulated during infection and inflammation. It is worth noting that CXCR2 is key stimulant of immune cells infiltration and recruitment, especially of neutrophils. Our results of evaluation of 22 subtypes immune cells infiltration showed that neutrophils are significantly elevated in AF patients compared with SR [52]. It is also reported that CXCR2 was involved in atrial monocytes infiltration, which accelerates atrial fibrosis and promotes atrial remodeling. Therefore, blocking *CXCR2* may serve as a new therapeutic strategy for AF patients [53]. Moreover, CXCR2 is also a crucial regulator of hypertension. In spontaneously hypertensive rats, Zhang et al*.* identified that inhibition of *CXCR2* could prevent the occurrence of hypertension-induced AF [53]. In angiotensin II-induced cardiac atrial fibrillation animal model, CXCR2 has also been proved to participate in immune cells infiltration and mediates cardiac hypertrophy and remodeling through regulation of monocyte [54].

S100A4, also known as fibroblast specific protein 1 (FSP1), is involved in various biological processes including cell survival, cell motility, and cell differentiation. Numerous studies have already revealed roles of S100A4 in cancer progression, particularly the ability of enhancing metastasis. S100A4 has also been linked to various diseases besides tumor, such as cardiac fibrosis and hypertrophy, kidney fibrosis and pulmonary disease. All of these diseases involve the inflammatory processes and rely heavily on tissue remodeling [55]. Studies revealed that *S100A4* expressed in normal human heart and increased in hypertrophic left ventricles [56–58]. In addition, S100A4 is a key regulator of endothelial mesenchymal transformation (EMT), which is related to immune cells infiltration, making epithelial cells present mesenchymal cell phenotype and ultimately resulting in enhanced migration ability, enhanced anti apoptosis ability and production of a large number of extracellular matrix components. Recently, studies demonstrated that EMT occurs in the atrium of AF patients and contribute to fibroblast accumulation. Meanwhile, S100A4 also had significant correlations with left atrial dimension in AF patients [59].

We used CIBERSORT to evaluate the degree of infiltrating immune cells in the present study. We found reduced infiltration of T cells follicular helper, as well as increased neutrophils, mast cells resting and T cells gamma delta in AF. Neutrophils represent activated nonspecific inflammation and have been found as markers of inflammation in various diseases. Correlation between inflammatory markers and cardiovascular diseases has been studied widely and the relationship between neutrophils and cardiovascular diseases has been confirmed in the past. It is reported that the level of neutrophils is an independent predictor for the prognosis of acute coronary syndrome [60]. Recently, accumulating studies have also reported that increased neutrophil/lymphocyte ratio is related to the increased risk of AF occurrence [61, 62]. Mast cells, tissue-specific innate immune cells, present in virtually all body tissues including the heart. Numerous inflammatory mediators secreted by mast cells including IL-1β, IL-6 and TGF-β1 participate in atrial structural remodeling and development of AF [63]. Liao et al*.* reported that the mast cells stabilization is associated with reduced atrial fibrosis and reduce AF incidence in animal model [64]. T follicular helper cells has also been reported to function in AF by secreting IL-21 and ultimately promoting B cell proliferation and differentiation, which might be activated by Toll-like receptor 2 (TLR2) and TLR4 [65, 66]. We also studied the correlation between *CYBB*, *CXCR2*, *S100A4* and infiltrating immune cells. Based on the correlation analysis results, *CYBB*, *CXCR2* and *S100A4* appear to play key roles in regulation of immune cells infiltration.

However, a limitation of the present study should be noted. The occurrence and development of atrial fibrillation is a complex and dynamic process, and its pathogenesis includes atrial electrical remodeling, atrial structural remodeling and autonomic nervous dysfunction. Although a total of 42 participants were included, the input data might still be insufficient to identify and validate key genes in the atrial fibrillation development. Moreover, the 42 participants included in the study came from various regions with different diet, physical activity, genetic variation, susceptibility to cardiovascular diseases, and so on. All of these factors may have an impact on atrial fibrillation. Therefore, the diagnostic efficacy of *CYBB*, *CXCR2*, and *S100A4* in different populations and its role in the occurrence of atrial fibrillation still need more external validation.

## Conclusions

We found that *CYBB*, *CXCR2* and *S100A4* may be key biomarkers of AF based on machine learning methods. The immune cells infiltration of patients with AF was measured in detail. Moreover, correlations between *CYBB*, *CXCR2* and *S100A4* and immune cells may play an important role in AF. Further researches for the specific molecular mechanism of these biomarkers and immune cells are required to study.

## Supplementary Information

**Additional file 1: Fig. S1**. PCA plot based on infiltrating immune cells of AF and SR atrial tissue samples. PCA, principal component analysis. AF, atrial fibrillation; SR, sinus rhythm.

**Additional file 2: Table S1**. Characteristics of the datasets included in the analysis.

## Data Availability

Publicly available datasets were analyzed in this study. All the raw data used in this study are derived from the public GEO data portal (https://www.ncbi.nlm.nih.gov/geo/).
