# Supplementary figures and images for: Analysis of potential genetic biomarkers using machine learning methods and immune infiltration regulatory mechanisms underlying atrial fibrillation
Source: BMC Med Genomics. 2022 Mar 19;15:64. doi: 10.1186/s12920-022-01212-0 (PMC8934464; doi:10.1186/s12920-022-01212-0)

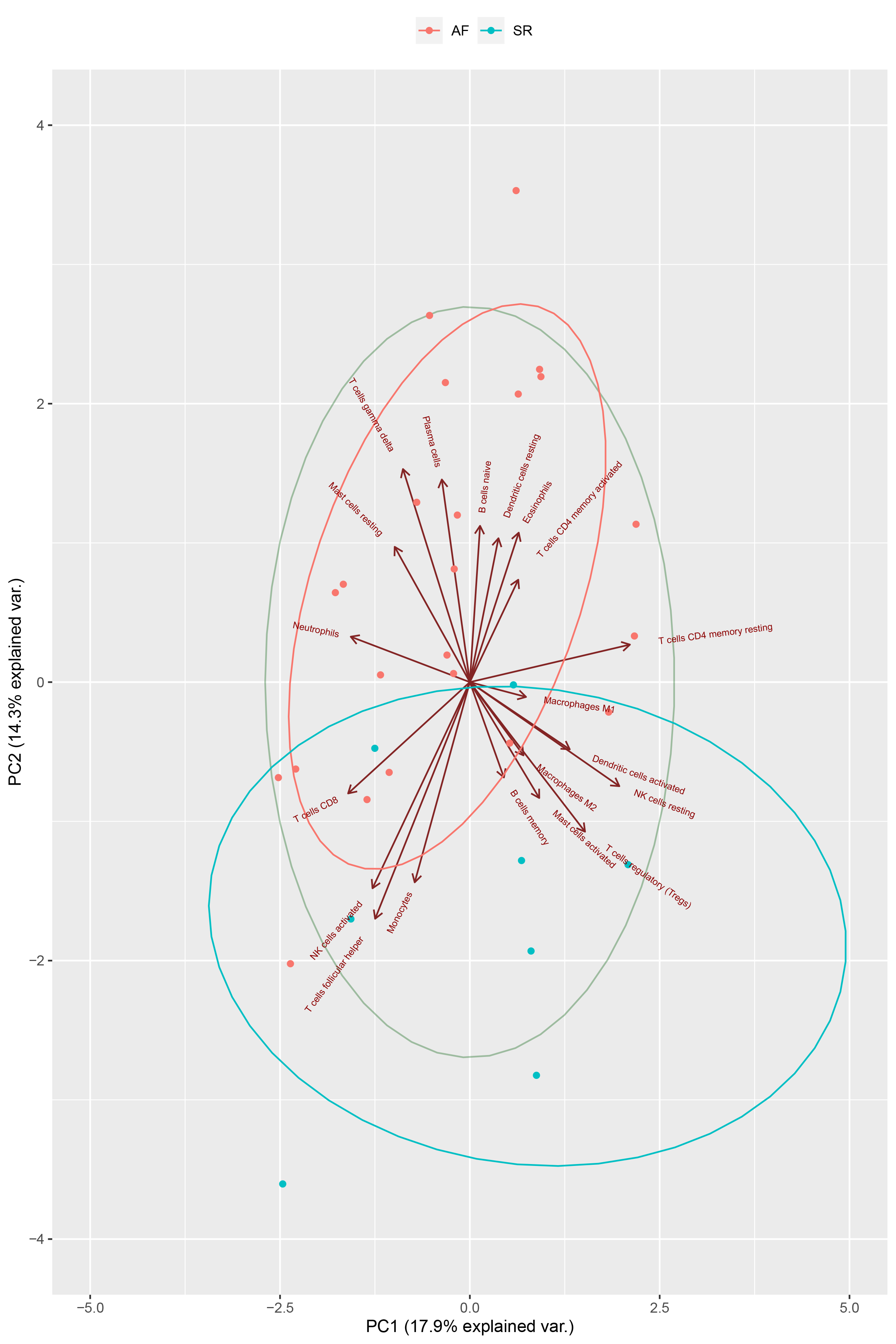

Supplement: Supplementary file 1 — Additional file 1: Fig. S1. PCA plot based on infiltrating immune cells of AF and SR atrial tissue samples. PCA, principal component analysis. AF, atrial fibrillation; SR, sinus rhythm. [file 12920_2022_1212_MOESM1_ESM.tif]
