# Supplementary material for: Analysis of potential genetic biomarkers using machine learning methods and immune infiltration regulatory mechanisms underlying atrial fibrillation
Source: BMC Med Genomics. 2022 Mar 19;15:64. doi: 10.1186/s12920-022-01212-0 (PMC8934464; doi:10.1186/s12920-022-01212-0)
Supplement: Supplementary file 2 — Additional file 2: Table S1. Characteristics of the datasets included in the analysis. [file 12920_2022_1212_MOESM2_ESM.docx]

**Table S1** Characteristics of the datasets included in the analysis.

| **GEO ID** | **Platform** | **Citation** | **Region** | **Role in this analysis** | **SR** | **AF** |
| --- | --- | --- | --- | --- | --- | --- |
| GSE41177 | GPL570; Affymetrix Human Genome U133 Plus 2.0 Array | Yeh YH, et al. Heart Rhythm, 2013;10(3):383-91. PMID: 23183193 | Taiwan, China | Training dataset | 3 | 16 |
| GSE79768 | GPL570; Affymetrix Human Genome U133 Plus 2.0 Array | Tsai FC, et al. Int J Cardiol, 2016;222:104-112. PMID: 27494721 | Taiwan, China | Training dataset | 6 | 7 |
| GSE14975 | GPL570; Affymetrix Human Genome U133 Plus 2.0 Array | Adam O, et al. J Am Coll Cardiol, 2010;55(5):469-80. PMID: 20117462 | Greifswald, Germany | Validation dataset | 5 | 5 |

GEO: Gene Expression Omnibus; SR: Sinus Rhythm; AF: Atrial Fibrillation.
